# Pepper RING‐type E3 ligase, CaANKR1, negatively regulates drought tolerance by modulating CaMAPK1 stability

**DOI:** 10.1111/tpj.71181

**Published:** 2026-10-14

**Authors:** Junyoung Cho, Chae Woo Lim, Bomyeon Yun, Dae Sung Kim, Sung Chul Lee

**Affiliations:** ^1^ Department of Life Science (BK21 Program) Chung‐Ang University Seoul 06974 Korea; ^2^ State Key Laboratory of Biocatalysis and Enzyme Engineering, School of Life Sciences Hubei University Wuhan 430062 China

**Keywords:** ABA, MAP kinase, E3 ligase, protein degradation, ubiquitination

## Abstract

Drought stress severely restricts plant growth and productivity, and abscisic acid (ABA)–mediated signaling plays a central role in coordinating adaptive responses to water deficit. Mitogen‐activated protein kinase (MAPK) cascades are widely involved in plant stress signaling; however, the ubiquitin‐mediated regulation of MAPK stability during drought responses remains poorly understood. In this study, we conducted a yeast two‐hybrid screening and identified CaMAPK1, a pepper (*Capsicum annuum*) MAPK, as an interacting partner of the RING‐type E3 ubiquitin ligase CaANKR1, which was further confirmed via *in vitro* and *in vivo* assays. *CaMAPK1* expression was induced by dehydration, osmotic stress, salinity stress, and ABA treatment. Furthermore, CaMAPK1 exhibited kinase activity and was predominantly localized to the nucleus. Ubiquitination assays revealed that CaANKR1 promoted K48‐linked polyubiquitination of CaMAPK1 in a RING domain–dependent manner. Consistently, cell‐free degradation assays showed that CaMAPK1 protein stability negatively correlated with *CaANKR1* expression levels, indicating that CaANKR1 promotes the proteasome‐dependent degradation of CaMAPK1. Phenotypic assays revealed that *CaMAPK1* silencing in pepper reduced drought tolerance by altering ABA‐mediated stomatal responses and water retention, whereas *CaMAPK1* overexpression in *Arabidopsis* produced the opposite phenotype. Collectively, these findings suggest that CaANKR1 negatively modulates ABA‐mediated drought tolerance through ubiquitin‐dependent destabilization of CaMAPK1. Together, they reveal a mechanism underlying ABA‐driven drought responses and highlight a promising target for enhancing crop drought resilience.

## Significance Statement

In our previous study, we elucidated that pepper RING‐type E3 ligase CaANRK1 functions as a negative modulator of ABA signaling and drought response. Here, we identified pepper MAP kinase CaMAPK1, which is ubiquitinated by CaANKR1 and positively modulates drought response via ABA‐mediated signaling.

## INTRODUCTION

Plants, as sessile organisms, are continuously exposed to various abiotic stresses, including drought, salinity, and extreme temperatures (Hirayama & Shinozaki, 2010; Zhu, 2016). Among these, drought is the most detrimental environmental factor, causing water deficit conditions that limit plant growth and ultimately reduce crop yield (Anjum et al., 2011; Ullah et al., 2017). In response to this, plants have evolved a wide range of physiological and molecular adaptations, including stomatal aperture regulation, water balance maintenance, and activation of stress‐responsive gene expression (Hu & Xiong, 2014; Yamaguchi‐Shinozaki & Shinozaki, 2006; Zhu, 2002). These adaptive responses are largely mediated by the phytohormone abscisic acid (ABA), which accumulates under drought conditions and functions as a central regulator of plant stress adaptation (Finkelstein et al., 2002; Golldack et al., 2014; Kong et al., 2025; Lv et al., 2026).

ABA signaling is initiated when ABA binds to PYRABACTIN RESISTANCE1/PYR1‐LIKE/REGULATORY COMPONENT OF ABA RECEPTOR (PYR/PYL/RCAR) receptors, which inhibit clade A protein phosphatase 2Cs (PP2Cs) and thereby release SNF1‐related protein kinases 2 (SnRK2s) for activation. This core PYR/PYL/RCAR‐PP2C‐SnRK2 module functions as a central regulatory hub that controls diverse downstream responses associated with drought stress (Cutler et al., 2010; Fujii et al., 2007; Park et al., 2009). Activated SnRK2s phosphorylate key downstream targets, such as ABF/AREB transcription factors, thereby promoting ABA‐responsive gene expression and stomatal regulation (Lee & Luan, 2012). Beyond this core pathway, ABA signaling is further modulated through extensive transcriptional and post‐translational regulatory networks.

Mitogen‐activated protein kinase (MAPK) cascades represent highly conserved signaling modules that mediate cellular responses to environmental and developmental cues (Colcombet & Hirt, 2008; Danquah et al., 2014). A canonical MAPK cascade comprises three sequentially acting kinases, MAPK kinase kinase (MAPKKK), MAPK kinase (MAPKK), and MAPK, where each tier activates the next through phosphorylation (Jonak et al., 2002). In *Arabidopsis thaliana*, approximately 20 MAPKs, 10 MAPKKs, and more than 60 MAPKKKs have been identified, highlighting the complexity and diversity of MAPK signaling networks in plants (Ichimura et al., 2002). Within this signaling module, MAPKs function as terminal kinases that phosphorylate diverse downstream targets, including transcription factors and stress‐responsive proteins (Colcombet & Hirt, 2008; Xu & Zhang, 2015). Growing evidence indicates that MAPK cascades participate in ABA signaling and drought stress responses. For example, *Arabidopsis* MPK9 and MPK12 regulate ABA‐dependent stomatal closure, while MPK1, MPK2, MPK7, and MPK14 are activated by ABA signaling (de Zelicourt et al., 2016). Similarly, maize ZmMPK3 is induced by ABA and drought‐related stresses (Wang et al., 2010). In contrast, wheat TaMPK3 negatively regulates ABA signaling by promoting the degradation of the ABA receptor TaPYL4, highlighting the functional diversity of MAPKs in stress signaling (Liu et al., 2022; Wang et al., 2010).

In addition to transcriptional control, ABA responses are extensively regulated through post‐translational modifications, which modulate protein activity, localization, and stability (Bae et al., 2024; Choi et al., 2025, 2026). Among these mechanisms, the ubiquitin–proteasome system (UPS) is a major pathway for selective protein degradation and plays a key role in plant growth, development, and stress adaptation (Sadanandom et al., 2012; Vierstra, 2009). Ubiquitination is mediated through the sequential action of three enzymes: ubiquitin‐activating enzymes (E1), ubiquitin‐conjugating enzymes (E2), and ubiquitin ligases (E3), with E3 ligases determining substrate specificity through direct target recognition (Moon et al., 2004). Really Interesting New Gene (RING)–type E3 ligases represent the largest E3 family in plants and have been widely implicated in diverse abiotic stress signaling, including ABA‐mediated responses (Bae et al., 2025; Baek et al., 2021; Koh et al., 2023). For example, *Arabidopsis* MAPKKK18 undergoes ubiquitin‐mediated degradation mediated by HECT‐ and RING‐type E3 ligases, thereby regulating ABA signaling (Tajdel‐Zielińska et al., 2024). However, despite the established roles of MAPK cascades in ABA‐mediated stress responses, their direct regulation by RING‐type E3 ligases under dehydration stress conditions remains largely unclear.

Previously, we reported that the RING‐type E3 ubiquitin ligase CaANKR1 functions as a negative regulator of ABA‐mediated drought stress responses in *Capsicum annuum* (Kim et al., 2025). In this study, we identified CaMAPK1 as an interacting partner of CaANKR1 and investigated its functional relationship with CaANKR1 in ABA‐mediated drought stress responses. We demonstrate that CaMAPK1 positively regulates ABA sensitivity and drought tolerance, while CaANKR1 promotes the ubiquitin‐mediated degradation of CaMAPK1 via the 26S proteasome pathway. Genetic and biochemical analyses revealed that CaMAPK1 functions downstream of CaANKR1 during ABA‐mediated drought responses. Collectively, these findings reveal a previously uncharacterized CaANKR1–CaMAPK1 regulatory module that fine‐tunes ABA‐mediated drought responses through ubiquitin‐dependent control of CaMAPK1 stability.

## RESULTS

### CaANKR1 physically interacts with CaMAPK1

In our previous study, CaANKR1 was characterized as a RING‐type E3 ubiquitin ligase that negatively regulates drought tolerance by modulating abscisic acid (ABA) signaling (Kim et al., 2025). To further elucidate the role of CaANKR1 in drought stress signaling, we aimed to identify interacting partners of CaANKR1. Y2H screening revealed potential interactions between CaANKR1 and several CaMAPKs (Figure S1A), among which CaMAPK1 exhibited a strong interaction and was therefore selected for subsequent functional and biochemical analyses. The interaction between CaANKR1 and CaMAPK1 was further confirmed using an individual Y2H assay (Figure 1A). Furthermore, a pull‐down assay verified the interaction between CaANKR1 and CaMAPK1 *in vitro* and revealed that GST–CaMAPK1 specifically pulled down MBP–CaANKR1, but not MBP alone (Figure 1B). This interaction was further validated *in vivo* via LCI (Figure 1C) and Co‐IP (Figure 1D) assays. Collectively, these results indicate that CaANKR1 physically interacts with CaMAPK1 both *in vivo* and *in vitro*.

**Figure 1:**
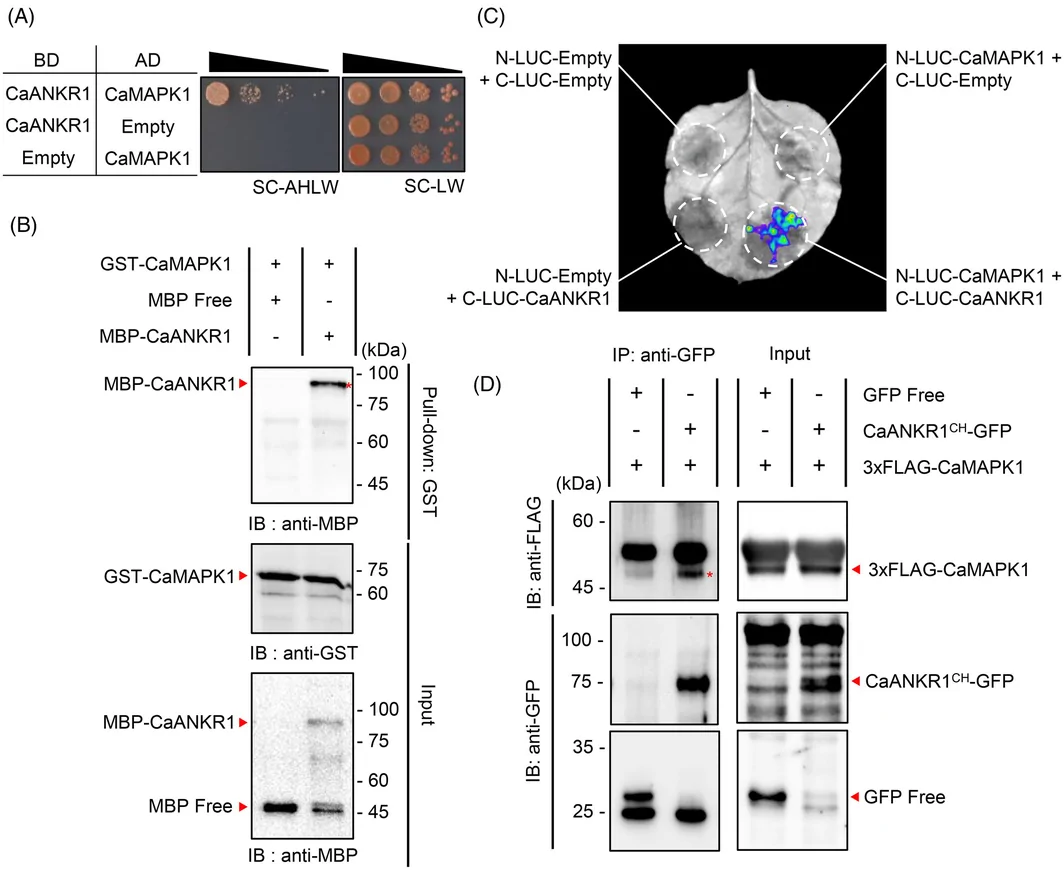
Physical interaction between CaANKR1 and CaMAPK1. (A) Yeast two‐hybrid assay of CaANKR1 and CaMAPK1. The Y2HGold strain was co‐transformed with BD‐CaANKR1 and AD‐CaMAPK1 and grown on SC–Leu–Trp (LW) as a control or SC–Ade–His–Leu–Trp (AHLW) media for selection. Serial dilutions (10^0^–10^−3^) were spotted and incubated at 28°C for 4 days. (B) *In vitro* pull‐down assay. GST–CaMAPK1 bound to glutathione resin was incubated with MBP–CaANKR1 or MBP (negative control). Bound proteins were analyzed by immunoblotting using anti‐GST and anti‐MBP antibodies. (C) Split‐luciferase complementation imaging assay. NLUC–CaMAPK1 and CLUC–CaANKR1 were co‐expressed in *Nicotiana benthamiana* leaves. After 3 days, 0.1 M D‐luciferin was infiltrated, and luminescence signals were captured using a CCD imaging system. Empty vector combinations served as negative controls. (D) *In vivo* co‐immunoprecipitation assay. CaANKR1^CH^‐GFP and 3×FLAG‐CaMAPK1 were co‐expressed in *N. benthamiana*. Proteins were immunoprecipitated with anti‐GFP magnetic beads and analyzed using anti‐FLAG and anti‐GFP antibodies. GFP protein was used as a negative control.

### Molecular characterization of CaMAPK1

Previous studies have shown that CaMAPK1 acts as a positive regulator of resistance to *Ralstonia solanacearum* infection and thermotolerance in pepper (Shi et al., 2024). To further characterize CaMAPK1, we first analyzed its expression patterns under various stress conditions using RT‐qPCR. Transcript levels were significantly induced by dehydration treatment, showing a marked increase at 6 h, followed by a partial decrease at 12 h, and reaching peak levels at 24 h. Under osmotic stress simulated by mannitol (600 mM), expression was strongly induced, peaking at 2 h, remaining relatively high at 6 h, and declining at 12 and 24 h. In response to NaCl (200 mM), transcript levels peaked at 6 h before gradually decreasing. In addition, ABA (100 μM) treatment markedly upregulated expression, with the highest induction observed at 12 h (Figure 2A). Next, organ‐specific expression levels of *CaMAPK1* were analyzed. *CaMAPK1* showed similar expression levels in leaves, stems, and roots, but relatively lower expression in flowers (Figure 2B). To determine the subcellular localization of CaMAPK1, GFP‐tagged CaMAPK1 was transiently expressed in the leaf epidermal cells of *Nicotiana benthamiana*. Two days after agroinfiltration, GFP signals from CaMAPK1 were predominantly detected in the nucleus (Figure 2C, left). This localization pattern did not appear to be independent of kinase activity, as the kinase‐dead mutant CaMAPK1^K72N^ exhibited the nuclear localization pattern (Figure 2C, right). To investigate the kinase activity of CaMAPK1, GFP‐tagged CaMAPK1 was transiently expressed in *N. benthamiana* leaves and immunoprecipitated using anti‐GFP magnetic beads. An *in vitro* kinase assay demonstrated that CaMAPK1 exhibited strong autophosphorylation activity, whereas CaMAPK1^K72N^ showed markedly reduced autophosphorylation activity (Figure 2D). Furthermore, an additional *in vitro* kinase assay using myelin basic protein (MBP) as an exogenous substrate demonstrated that CaMAPK1 phosphorylated MBP, whereas CaMAPK1^K72N^ showed no detectable substrate phosphorylation activity (Figure 2E).

**Figure 2:**
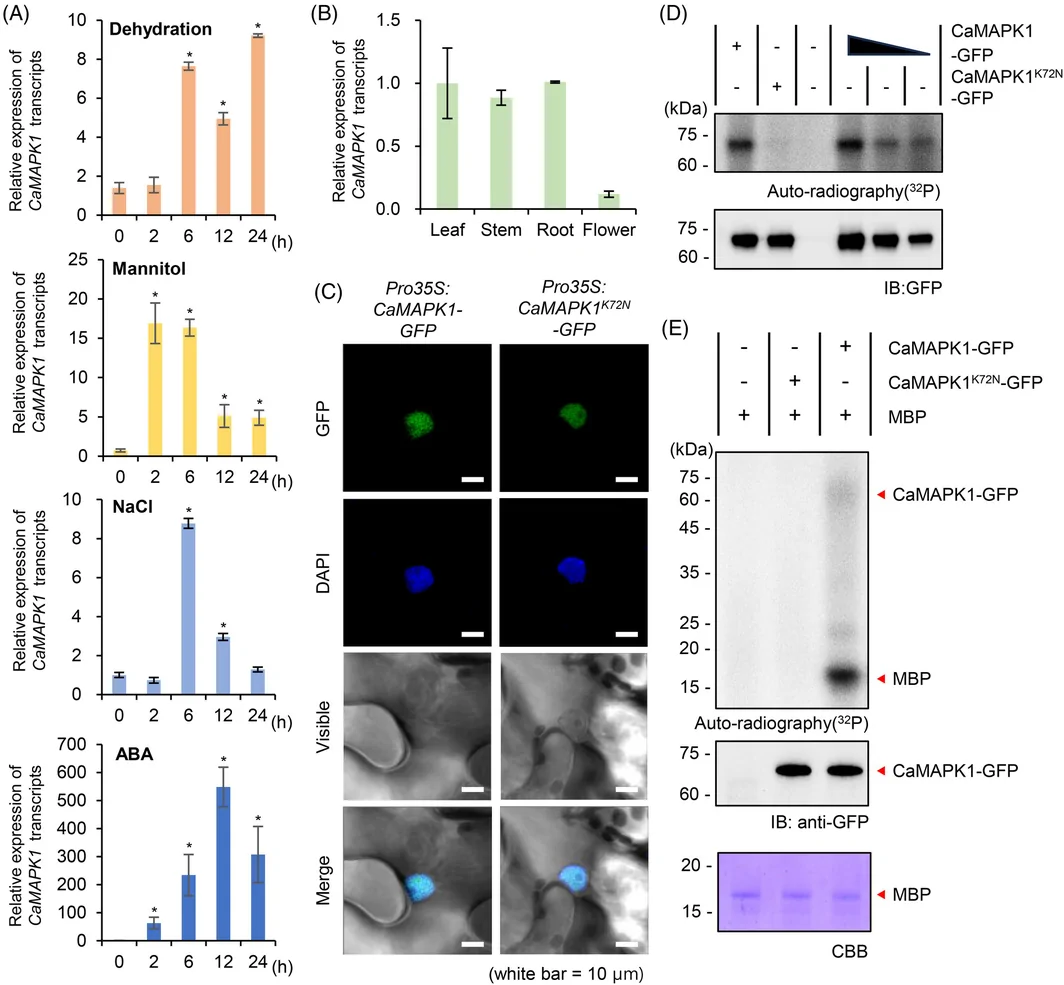
Molecular characterization of CaMAPK1. (A) Expression patterns of *CaMAPK1* in pepper leaves under dehydration, 600 mM mannitol, 200 mM NaCl, and 100 μM abscisic acid (ABA) treatments. Samples were collected at 0, 2, 6, 12, and 24 h after treatment. Transcript levels were analyzed using RT‐qPCR and normalized to *CaACT1*. Expression levels at 0 h were set to 1.0. Values represent the means ± standard error of three independent experiments. Asterisks indicate significant differences compared with the control (0 h) (Student's *t*‐test; *P < 0.05). (B) Organ‐specific expression of *CaMAPK1* in leaf, stem, root, and flower tissues. Relative expression levels (ΔΔCT) were normalized to *CaACT1*, and expression in leaf tissue was set to 1.0. (C) Subcellular localization of CaMAPK1 and CaMAPK1^K72N^ (kinase‐dead mutant). CaMAPK1‐GFP and CaMAPK1^K72N^‐GFP were transiently expressed in *Nicotiana benthamiana* leaves via agroinfiltration and observed using confocal microscopy. 4′,6‐Diamidino‐2‐phenylindole (DAPI) was used as a nuclear marker. (D) *In vitro* kinase assay of CaMAPK1. CaMAPK1–GFP and CaMAPK1^K72N^–GFP were transiently expressed in *N. benthamiana* leaves. GFP‐tagged proteins were immunoprecipitated using anti‐GFP magnetic beads and incubated with [γ‐^32^P]‐ATP. Phosphorylation was detected using ^32^P autoradiography. (E) *In vitro* kinase assay of CaMAPK1 using myelin basic protein (MBP) as an exogenous substrate. Immunoprecipitated CaMAPK1–GFP and CaMAPK1^K72N^–GFP were incubated with MBP in the presence of [γ‐^32^P]‐ATP. Phosphorylation signals were detected by ^32^P autoradiography. Protein levels were verified by immunoblot analysis using an anti‐GFP antibody and MBP loading was visualized by Coomassie Brilliant Blue staining.

### Silencing of *CaMAPK1* enhances drought sensitivity in pepper

To examine the biological role of CaMAPK1 in pepper, we generated *CaMAPK1*‐silenced pepper using a tobacco rattle virus (TRV)‐based VIGS system. RT‐PCR analysis verified that *CaMAPK1* transcript levels were significantly reduced in TRV2:*CaMAPK1* plants compared with those in TRV2:00 (control) (Figure 3A). Given that *CaMAPK1* expression was induced by dehydration and ABA treatment, we confirmed whether CaMAPK1 is involved in drought stress responses. To test this possibility, TRV2:*CaMAPK1* and TRV2:00 plants were subjected to drought stress by withholding water for 14 days, followed by rewatering for 3 days (Figure 3B). Under well‐watered and drought conditions, no obvious phenotypic differences were observed between TRV2:*CaMAPK1* and TRV2:00 plants (Figure 3B, upper and middle panels). However, after rewatering, *CaMAPK1*‐silenced plants exhibited more wilting and a lower survival rate than those in control plants (Figure 3B, lower panel). To confirm whether this phenotype was associated with differences in water retention capacity, we monitored transpirational water loss by measuring the fresh weight of detached first and second leaves at the indicated time points (Figure 3C). As expected, we observed a greater reduction in the fresh weight of TRV2:*CaMAPK1* leaves than in TRV2:00 leaves. Given that stomatal regulation is a major determinant of transpirational water loss and is primarily regulated by ABA, we further evaluated ABA‐induced responses by analyzing leaf temperature and stomatal aperture. Under ABA treatment, TRV2:*CaMAPK1* plants exhibited lower leaf surface temperatures and larger stomatal apertures than did TRV2:00 plants, whereas no significant differences were observed under normal conditions (Figure 3D–F). Collectively, these results suggest that CaMAPK1 functions as a positive regulator of drought tolerance in pepper by facilitating ABA‐mediated stomatal closure.

**Figure 3:**
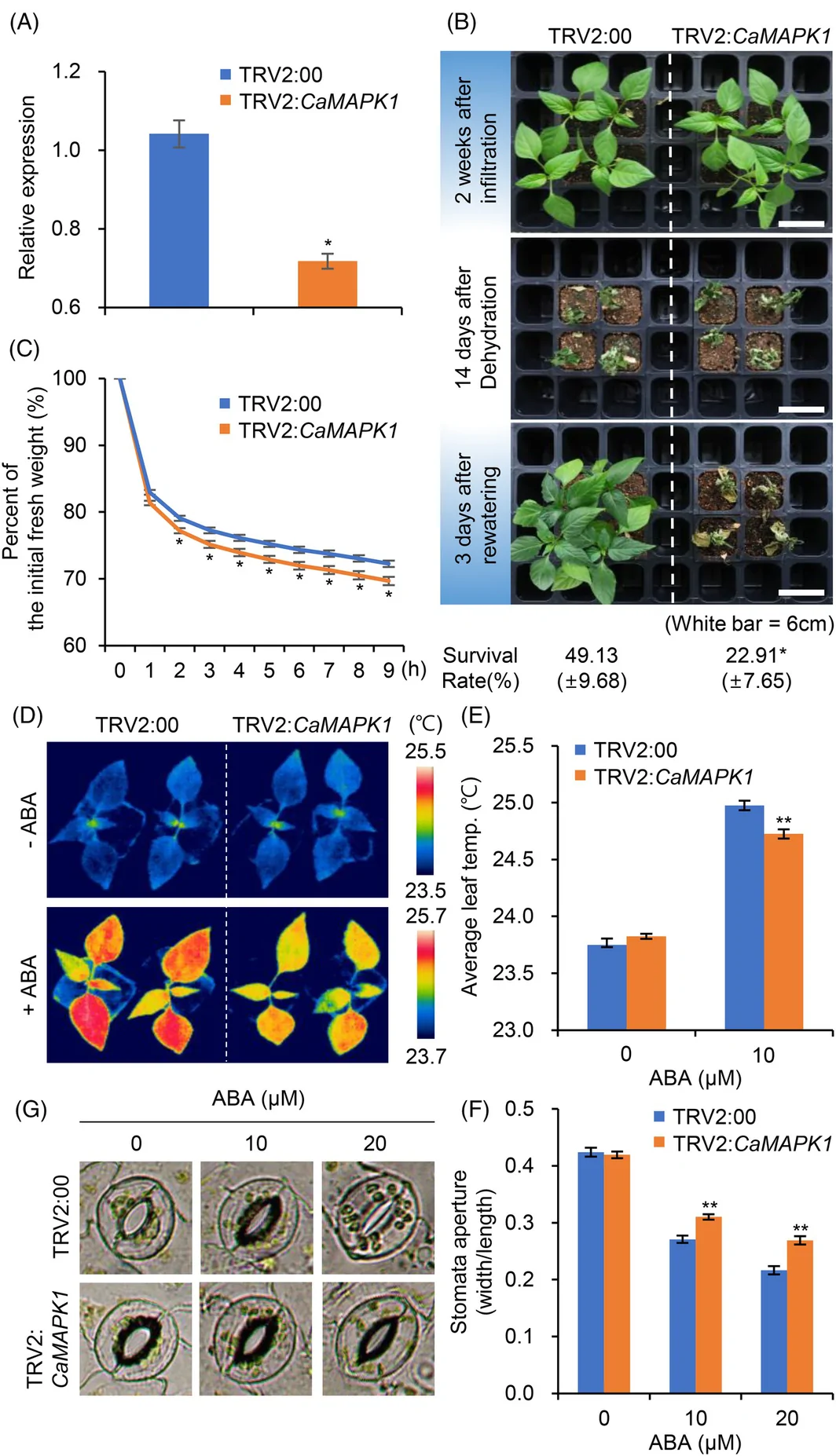
Reduced drought tolerance in *CaMAPK1*‐silenced pepper. (A) RT‐PCR analysis of *CaMAPK1* expression levels in control (TRV2:00) and *CaMAPK1*‐silenced (TRV2:*CaMAPK1*) pepper. *CaACT1* was used as an internal control. (B) Drought sensitivity of TRV2:00 and TRV2:*CaMAPK1* pepper. Three‐week‐old plants were subjected to drought stress by withholding watering for 14 days, followed by rewatering for 3 days. Representative images were acquired after rewatering (*n* = 36 plants per genotype). (C) Transpirational water loss from detached leaves of TRV2:00 and TRV2:*CaMAPK1* pepper. Leaf fresh weights were measured at the indicated time points and expressed as a percentage of the initial weight (*n* = 10 leaves per genotype). (D, E) Leaf temperature of TRV2:00 and TRV2:*CaMAPK1* plants after treatment with 100 μM ABA. Quantification of mean leaf surface temperatures measured from the first and second leaves of each plant (*n* = 10–20 plants per genotype). (F, G) Stomatal apertures (width‐to‐length ratio) of TRV2:00 and TRV2:*CaMAPK1* pepper in response to 0, 10, or 20 μM ABA treatment. At least 100 stomata were analyzed per genotype. Data represent mean ± SE from three independent experiments. Asterisks indicate significant differences (*P < 0.05, **P < 0.01; Student's *t*‐test).

### *CaMAPK1* overexpression enhances drought tolerance in *Arabidopsis*

Two independent transgenic *Arabidopsis* lines *CaMAPK1*‐OX revealed the biological role of *CaMAPK1* in ABA signaling and drought responses. RT‐PCR analysis confirmed that *CaMAPK1* transcript levels were higher in the OX lines (#6 and #9) than in wild‐type (Col‐0) plants (Figure 4A). The effect of *CaMAPK1* overexpression on ABA sensitivity during seed germination and seedling development was evaluated by assessing the plant responses in the presence or absence of ABA. Under normal conditions, no significant differences were observed between wild‐type and *CaMAPK1*‐OX lines. However, in the presence of 0.3 μM ABA, *CaMAPK1*‐OX lines exhibited reduced germination rates (Figure 4B), suppressed cotyledon greening (Figure 4C), and inhibited primary root elongation (Figure 4D,E) than those in wild‐type plants. To further evaluate ABA sensitivity in *CaMAPK1*‐OX plants, post‐germinative growth assays were conducted by transferring 3‐day‐old seedlings to 1/2 MS medium containing 0, 5, or 10 μM ABA. Primary root growth was comparable between the wild‐type and *CaMAPK1*‐OX lines in the absence of ABA. However, under ABA treatment, the *CaMAPK1*‐OX lines exhibited shorter primary roots than wild‐type plants (Figure S2A,B), indicating enhanced ABA sensitivity during post‐germinative root growth. In addition, to assess the response of *CaMAPK1*‐OX plants to osmotic stress during germination and early seedling development, seeds were germinated on 1/2 MS medium supplemented with 0 or 400 mM mannitol. Under mannitol treatment, the *CaMAPK1*‐OX lines exhibited lower germination rates and reduced green cotyledon development than wild‐type plants, whereas no significant differences were observed under control conditions (Figure S2C,D). Given that *CaMAPK1*‐OX lines exhibit enhanced sensitivity to ABA during early developmental stages, we further examined whether this ABA‐sensitive phenotype influences drought tolerance at the adult stage. Under well‐watered and drought conditions, no visible differences were observed between wild‐type and *CaMAPK1*‐OX lines (Figure 4F, upper and middle panels). However, after 14 days of water withholding followed by 3 days of rewatering, *CaMAPK1*‐OX lines exhibited less wilting and higher survival rates than wild‐type plants (Figure 4F, lower panel). To investigate whether this phenotype is associated with changes in water retention capacity, we measured transpirational water loss using detached shoots. *CaMAPK1*‐OX lines showed a slower reduction in fresh weight than wild‐type plants, indicating reduced water loss (Figure 4G). ABA‐mediated stomatal responses were assessed by measuring leaf surface temperature and stomatal aperture. Consistently, *CaMAPK1*‐OX lines exhibited higher leaf surface temperatures and smaller stomatal apertures under ABA treatment (Figure 4H–J). These results indicate that CaMAPK1 positively regulates ABA‐mediated drought responses in *Arabidopsis* plants.

**Figure 4:**
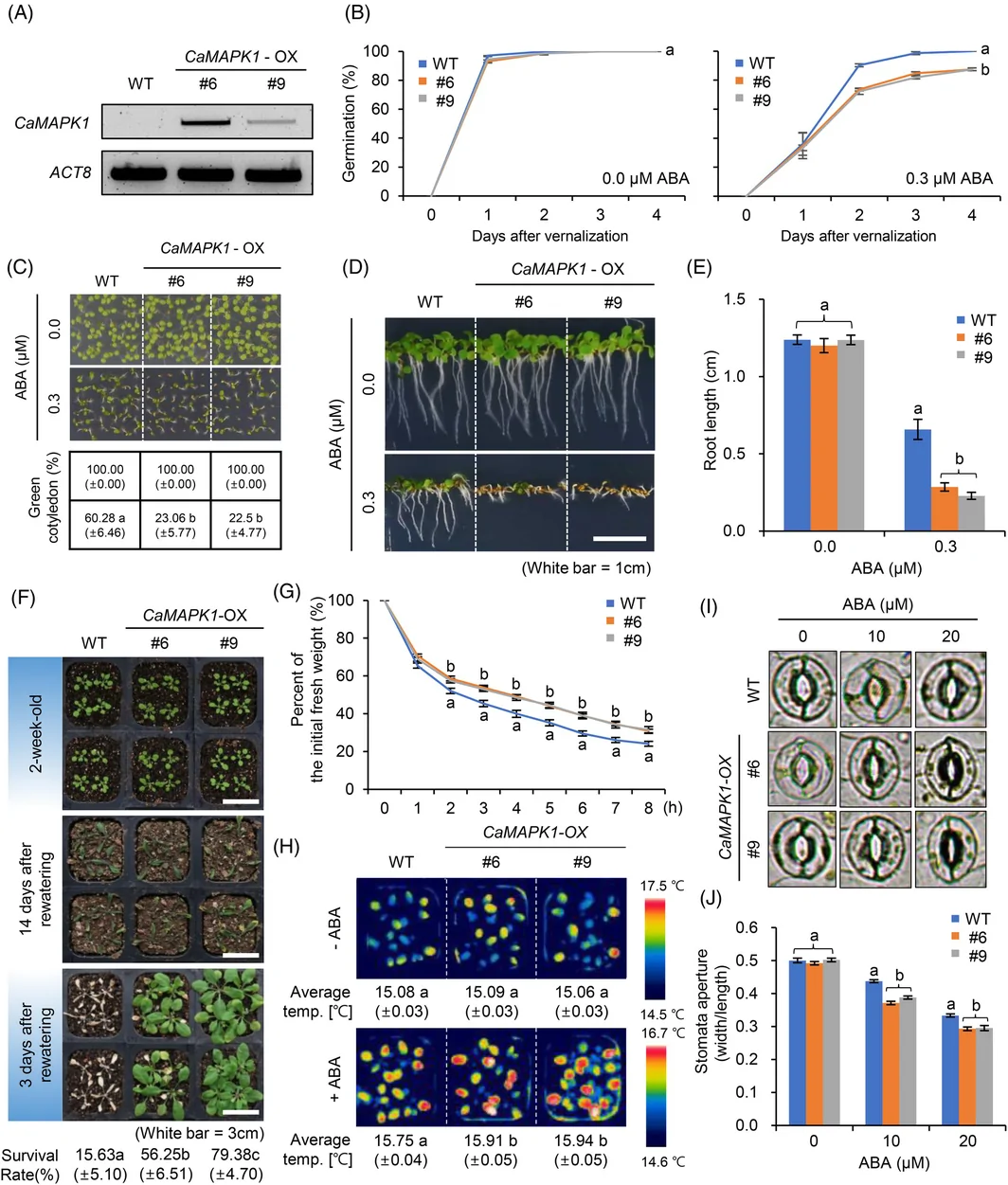
Increased ABA sensitivity and drought tolerance of *CaMAPK1*‐OX *Arabidopsis* plants. (A) RT‐PCR analysis of *CaMAPK1* expression in Col‐0 [wild‐type (WT)] and *CaMAPK1*‐OX *Arabidopsis* lines. *Actin8* was used as an internal control. (B) Germination rates of WT and transgenic lines in response to ABA. Seeds of each line were sown on 1/2‐strength Murashige and Skoog (1/2 MS) agar plates containing either 0 or 0.3 μM ABA. Germination was scored by counting seeds showing radicle emergence at the indicated time points after plating. (C) Cotyledon greening of WT and transgenic lines in response to ABA. Seeds were germinated on 1/2 MS agar plates supplemented with 0 or 0.3 μM ABA. Seven days after planting, the percentage of seedlings with green cotyledons was determined (*n* = 72 seeds per genotype). (D, E) Primary root growth of WT and transgenic lines in response to ABA. Seeds of each line were sown on 1/2 MS agar plates containing 0 or 0.3 μM ABA. Plates were placed vertically for 5 days. Representative images were obtained (D), and primary root length was measured in (E) (*n* = 20 seedlings per genotype). (F) Drought phenotype of WT and transgenic lines. Three‐week‐old plants grown under well‐watered conditions were subjected to drought stress by withholding water for 14 days, followed by rewatering for 3 days. Survival rates were calculated after rewatering (*n* = 48 plants per genotype). (G) Transpirational water loss from the detached leaves of WT and transgenic lines. Detached leaves were weighed at the indicated time points after detachment, and water loss was expressed as a percentage of the initial fresh weight (*n* = 10 leaves per genotype). (H) Leaf surface temperature of WT and transgenic lines after ABA treatment. Thermal images were obtained 3 h after treatment with 100 μM ABA, and leaf temperatures were measured using thermal imaging (*n* = 32 plants per genotype). (I, J) Stomatal aperture of WT and transgenic lines in response to ABA treatment. Epidermal peels were treated with 0, 10, or 20 μM ABA, and stomatal apertures were measured. Stomatal images are shown in (I), and quantification of stomatal aperture (width‐to‐length ratio) is presented in (J). At least 100 stomata were analyzed per genotype. All data represent the mean ± SE of three independent biological replicates. Statistical analyses were performed using two‐way ANOVA followed by Tukey's post hoc test (*P* < 0.05). Different letters indicate significant differences among genotypes within the same treatment, whereas genotypes sharing the same letter are not significantly different.

### CaANKR1 regulates CaMAPK1 stability via ubiquitination

To investigate whether CaMAPK1 is regulated through the ubiquitin–proteasome system, we performed an *in vivo* protein stability assay. GFP‐tagged CaMAPK1 was transiently expressed in *N. benthamiana* leaves, and cycloheximide (CHX) was infiltrated in the presence or absence of MG132. Immunoblot analysis using an anti‐GFP antibody showed that MG132 treatment led to the accumulation of CaMAPK1 and induced a band shift. In addition, ubiquitination of CaMAPK1 was confirmed using an anti‐UBQ1 antibody (Figure S3A). These results indicate that CaMAPK1 undergoes ubiquitin‐dependent degradation via the 26S proteasome pathway, suggesting that its stability is subject to ubiquitin‐mediated regulation.

Given that CaMAPK1 undergoes ubiquitin‐dependent degradation, we sought to determine whether CaANKR1, a RING‐type E3 ubiquitin ligase, might be involved in regulating CaMAPK1 stability. To test this hypothesis, an *in vitro* ubiquitination assay was performed using GST‐CaMAPK1 as the substrate. A K48‐linked polyubiquitin signal was detected only in the complete reaction containing ubiquitin, UBE1 (E1), UbcH5b (E2), MBP‐CaANKR1, and GST‐CaMAPK1 (Figure 5A, lane 5). Omission of E1, E2, or MBP‐CaANKR1 abolished the K48‐linked polyubiquitin signal (Figure 5A, lanes 2–4). Consistently, replacement of MBP‐CaANKR1 with the RING‐dead mutant MBP‐CaANKR1^CH^ (H338Y/C341S) did not produce a comparable K48‐linked polyubiquitin signal (Figure 5A, lane 6). The faint band detected near the molecular weight of MBP‐CaANKR1^CH^ was also present in the control reaction lacking ubiquitin, E1, and E2 (Figure 5A, lane 7), indicating that it does not represent a ubiquitination product. Furthermore, immunoblot analyses using anti‐GST and anti‐MBP antibodies demonstrated ubiquitination‐dependent mobility shifts and high‐molecular‐weight smears of GST‐CaMAPK1 and MBP‐CaANKR1 only in the complete reaction. These results indicate that CaANKR1 catalyzes K48‐linked ubiquitination of CaMAPK1 *in vitro*. Based on these data, we confirmed whether CaANKR1‐mediated ubiquitination affects CaMAPK1 stability through a cell‐free degradation assay using leaf crude extracts from *CaANKR1*‐silenced pepper or *CaANKR1*‐OX *Arabidopsis*. Using GST–CaMAPK1 as the substrate, reduced degradation was observed after incubation with crude extracts from pTRV2:*CaANKR1*‐silenced pepper compared with that in pTRV2:00 control plants (Figure 5B). In contrast, enhanced degradation was observed in extracts from two independent *CaANKR1*‐OX *Arabidopsis* lines (#10 and #14) compared with that in wild‐type plants (Figure 5C). Furthermore, CaMAPK1 was degraded more rapidly in extracts from *CaANKR1*‐OX lines than in those from *CaANKR1* ^CH^‐OX lines, indicating that the E3 ligase activity of CaANKR1 promotes CaMAPK1 degradation (Figure S4B).

**Figure 5:**
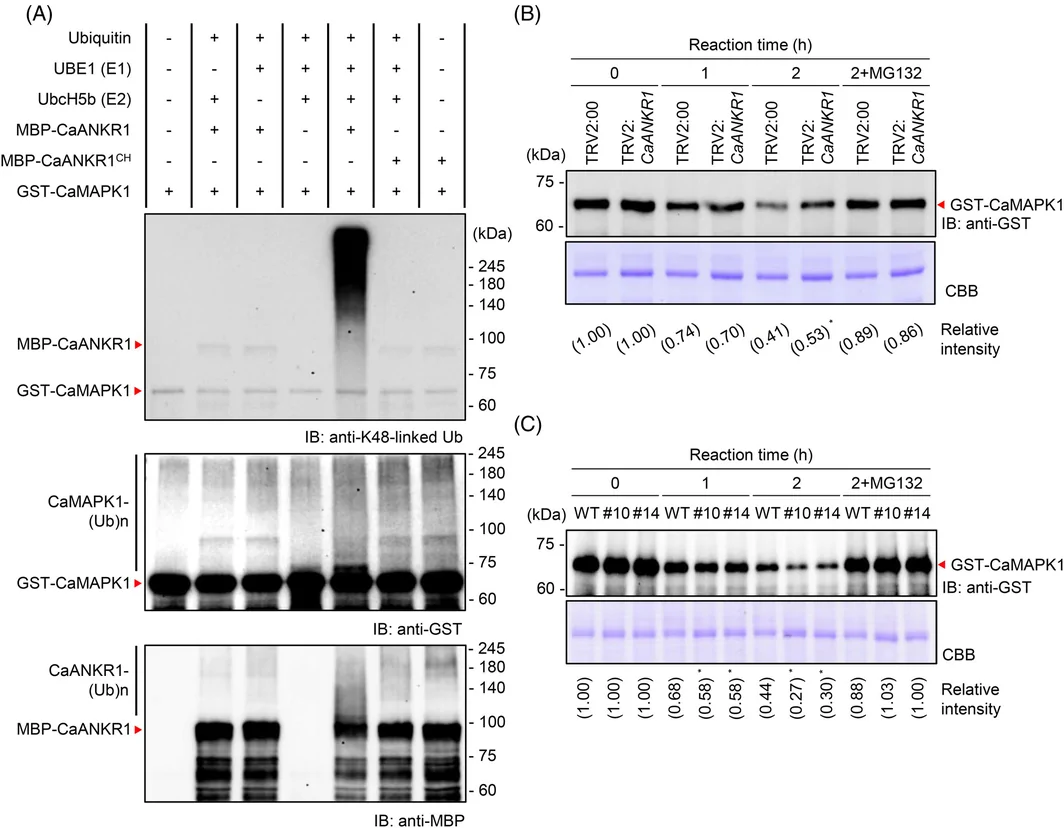
CaANKR1‐mediated regulation of CaMAPK1 protein stability. (A) *In vitro* ubiquitination assay of CaMAPK1 by CaANKR1. Equal amounts of GST‐tagged CaMAPK1 were incubated with the indicated combinations of ubiquitin, UBE1 (E1), UbcH5b (E2), MBP–CaANKR1, and the RING‐dead mutant MBP–CaANKR1^CH^. Reactions lacking the indicated components, including GST–CaMAPK1 alone or together with MBP–CaANKR1^CH^, were included as negative controls. Immunoblot analyses were performed using anti‐K48‐linked ubiquitin (Ub) (upper panel), anti‐GST (middle panel), and anti‐MBP (lower panel) antibodies. (B, C) Cell‐free degradation assay for CaMAPK1. GST–CaMAPK1 protein was incubated with crude extracts prepared from the leaves of control (TRV:00) or *CaANKR1*‐silenced pepper (TRV2:*CaANKR1*) (B), and from wild‐type (WT) or *CaANKR1*‐OX *Arabidopsis* plants (#10, #14) (C) for 0, 1, or 2 h with MG132 treatment at 2 h. Immunoblot analysis was performed using anti‐GST antibody (top), and Coomassie Brilliant Blue staining indicates equal loading of crude extract (bottom). The relative intensities of GST–CaMAPK1 fusion proteins were quantified using ImageJ software (version 1.52a). Protein levels were normalized to the initial level (0 h), which was set to 1.0. Statistical significance was determined using Student's *t*‐test (*P* < 0.05).

### CaMAPK1 functions downstream of CaANKR1 in drought stress responses

To determine the genetic relationship between CaANKR1 and CaMAPK1 in drought stress responses, we generated double‐silenced pepper along with the corresponding single‐silenced lines, as previously described (Bae et al., 2025; Choi et al., 2026). The transcript levels of each gene were significantly reduced in the corresponding silenced plants (Figure S5A). Under drought stress conditions, *CaANKR1*‐silenced plants exhibited enhanced tolerance, whereas *CaMAPK1*‐silenced plants showed increased sensitivity compared with those in control plants. Notably, the double‐silenced plants displayed a drought‐sensitive phenotype similar to that of *CaMAPK1*‐silenced plants, with reduced survival rates after rewatering (Figure 6A,B). Consistently, both transpirational water loss and leaf temperature responses followed the same pattern, with the responses of double‐silenced plants comparable to those of *CaMAPK1*‐silenced plants (Figure 6C–E). Taken together, these results indicate that CaMAPK1 functions downstream of CaANKR1 in regulating ABA‐mediated drought responses.

**Figure 6:**
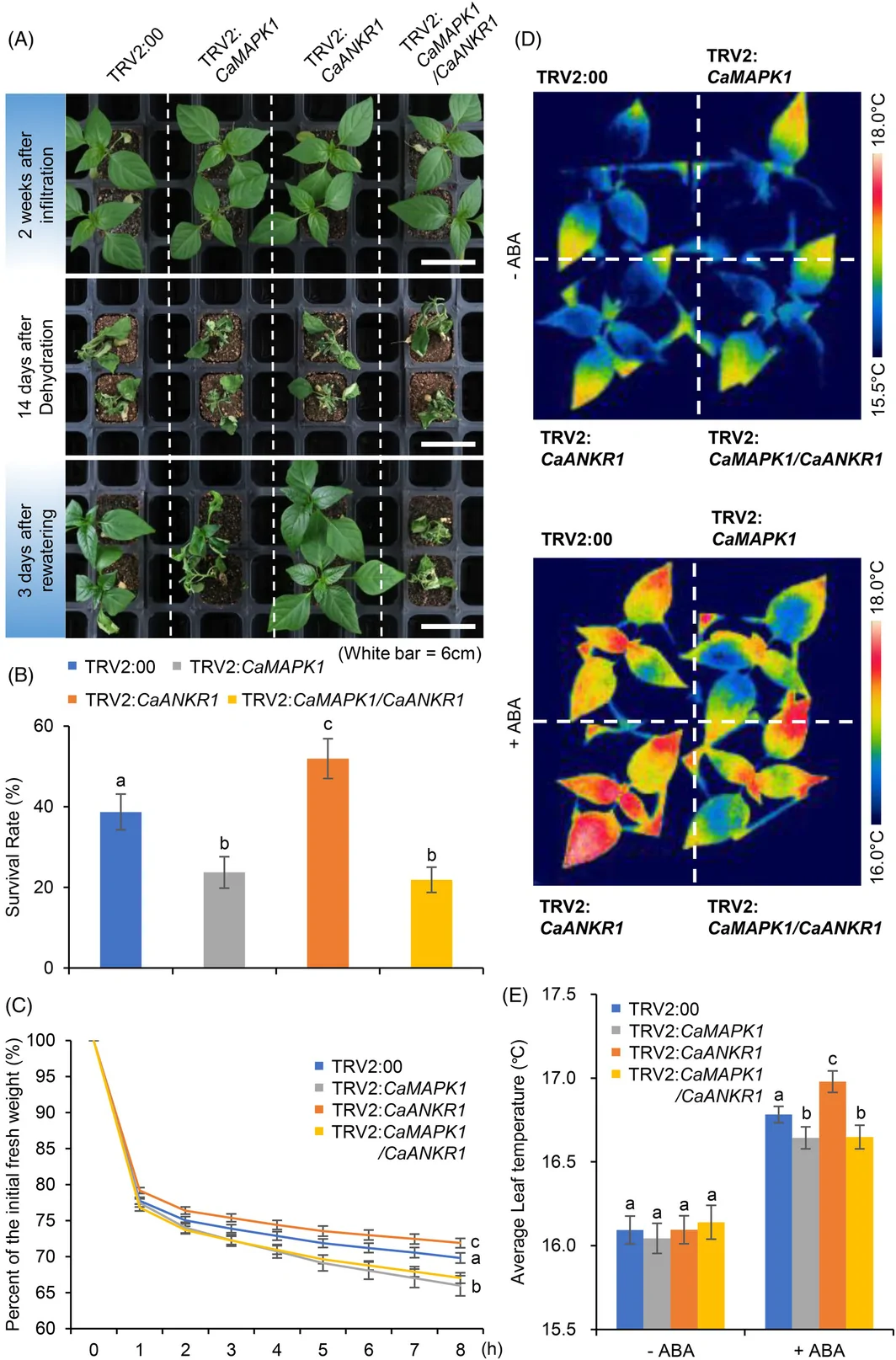
Genetic relationship between CaANKR1 and CaMAPK1 in drought stress responses. (A) Reduced drought tolerance of *CaMAPK1*/*CaANKR1* double gene–silenced pepper. Two weeks after agroinfiltration, plants were subjected to drought stress by withholding water for 14 days, followed by rewatering for 3 days. Representative images were obtained before drought treatment (top), after drought stress (middle), and after rewatering (bottom). (B) Survival rate of pepper after rewatering. (C) Transpirational water loss from detached leaves of TRV:00, TRV:*CaMAPK1*, TRV:*CaANKR1*, and TRV:*CaMAPK1*/*CaANKR1* pepper. Leaf fresh weights were measured at the indicated time points after detachment. (D, E) Leaf temperatures of TRV:00, TRV:*CaMAPK1*, TRV:*CaANKR1*, and TRV:*CaMAPK1*/*CaANKR1* pepper 3 h after treatment with 100 μM ABA. Representative thermographic images are shown in (D), and the quantification of leaf temperatures is shown in (E). All data represent the mean ± SE of three independent biological replicates. Statistical analyses were performed using two‐way ANOVA followed by Tukey's post hoc test (*P* < 0.05). Different letters indicate significant differences among genotypes within the same treatment, whereas genotypes sharing the same letter are not significantly different.

## DISCUSSION

Our findings demonstrate that the RING‐type E3 ligase, CaANKR1, negatively regulates ABA‐mediated drought responses by controlling CaMAPK1 stability through ubiquitin‐mediated proteasomal degradation. Protein ubiquitination and proteasomal degradation are widely recognized as critical post‐translational regulatory mechanisms in plant responses to environmental stresses (Bachmair et al., 2001; Belknap & Garbarino, 1996; Stone, 2019). E3 ubiquitin ligases confer substrate specificity in this pathway, and therefore, serve as key determinants of protein stability under stress conditions (Shu & Yang, 2017; Smalle & Vierstra, 2004; Vierstra, 2009). Among the diverse classes of E3 ligases, RING‐type E3 ligases constitute one of the largest groups in plants and have been intensively studied for their roles in abiotic stress responses (Cho et al., 2017; Stone et al., 2005). Previous studies have reported that various RING‐type E3 ligases modulate dehydration and ABA responses either positively or negatively depending on their target substrates. For example, SDIR1 positively regulates ABA‐mediated drought responses in *Arabidopsis*, as its overexpression enhances ABA sensitivity, ABA‐induced stomatal closure, and drought tolerance (Zhang et al., 2007). In contrast, CaFIRF1 negatively regulates dehydration stress tolerance in pepper by promoting ubiquitin‐mediated degradation of the positive regulator CaSAP14 (Bae et al., 2025). Nevertheless, despite their importance, how RING‐type E3 ligases control the stability of specific drought‐responsive signaling proteins remains only partially understood. In particular, our results identify CaMAPK1 as a direct ubiquitination target of CaANKR1 during ABA‐mediated drought signaling.

MAPKs have been widely implicated in plant responses to abiotic stresses, including drought, osmotic stress, salinity, and ABA signaling (Danquah et al., 2014; de Zelicourt et al., 2016). Consistent with this, *CaMAPK1* transcript levels were strongly induced by dehydration, mannitol, NaCl, and ABA treatments (Figure 2A), indicating that CaMAPK1 is involved in multiple stress‐responsive signaling pathways. Both loss‐ and gain‐of‐function analyses revealed that CaMAPK1 positively regulates ABA‐mediated drought stress responses. *CaMAPK1*‐silenced pepper exhibited reduced drought tolerance, characterized by lower survival rates after rewatering and increased transpirational water loss. Following ABA treatment, these plants also showed decreased leaf surface temperatures and larger stomatal apertures (Figure 3). Conversely, *CaMAPK1*‐OX *Arabidopsis* plants displayed enhanced ABA sensitivity during seed germination, cotyledon greening, and primary root growth, as well as improved drought tolerance at the adult stage (Figure 4). These drought‐tolerant phenotypes exhibited reduced water loss, elevated leaf surface temperatures after ABA treatment, and smaller stomatal apertures compared with those in wild‐type plants. Collectively, these findings indicate that CaMAPK1 positively regulates ABA‐associated drought stress responses by enhancing ABA‐dependent stomatal regulation.

Notably, CaMAPK1 expression reached peak induction at 24 h of dehydration, suggesting its specific role in the late phase of stress signaling. In *Arabidopsis*, the MAP3K17/18–MKK3–MPK1/2/7/14 cascade has been proposed to regulate late ABA‐induced gene expression, indicating that MAPK modules can participate in delayed ABA responses (de Zelicourt et al., 2016). This module is activated, at least in part, through ABA‐induced transcriptional upregulation of MAP3K17/18 and subsequent MAP3K protein synthesis, linking MAPK signaling to the later phase of ABA‐responsive signaling. Considering its high sequence homology with *Arabidopsis* MPK1 and MPK2, CaMAPK1 likely participates in sustained ABA‐responsive signaling rather than functioning exclusively during early ABA signaling. Such delayed activation may sustain ABA signaling during prolonged dehydration stress while avoiding excessive inhibition of early growth responses.

While MAPK signaling is primarily regulated by phosphorylation, recent studies indicate that MAPK cascade components are also controlled by ubiquitination‐mediated protein turnover, including regulation by RING‐type E3 ligases. For example, in *Arabidopsis*, the RING‐type E3 ligase KEG ubiquitinates MKK4 and MKK5 to regulate MAPK‐mediated immune responses (Gao et al., 2021). Moreover, KEG, together with the HECT‐type E3 ligases UPL1 and UPL4, promotes the turnover of MAPKKK17/18, which are involved in ABA signaling, drought tolerance, and senescence (Tajdel‐Zielińska et al., 2024). In addition, the RING‐type E3 ligases RGLG1 and RGLG2 ubiquitinate MAPKKK18 and negatively regulate MAPKKK18‐mediated drought stress tolerance (Yu et al., 2021). However, compared with MAPKKKs and MAPKKs, direct ubiquitination‐mediated regulation of MAPKs by RING‐type E3 ligases during ABA‐mediated drought responses remains largely unexplored. Our findings also highlight the importance of ubiquitin‐mediated turnover in MAPK signaling. We demonstrated that CaANKR1 promotes the K48‐linked ubiquitination of CaMAPK1 in a RING domain–dependent manner (Figure 5A). Cell‐free degradation assays confirmed that CaMAPK1 stability is inversely correlated with CaANKR1 levels (Figure 5B,C), indicating that CaANKR1 negatively regulates CaMAPK1 accumulation. This regulatory axis likely prevents excessive ABA signaling that could compromise plant growth under prolonged stress conditions.

While this study establishes the CaANKR1–CaMAPK1 regulatory module, several questions remain to be addressed. The precise downstream targets of CaMAPK1 in the nucleus remain to be identified. Although direct evidence linking the CaANKR1–CaMAPK1 module to late ABA‐responsive gene expression is still lacking, given its nuclear localization and kinase activity, CaMAPK1 likely phosphorylates specific transcription factors that orchestrate the late ABA‐responsive transcriptome. Furthermore, exploring whether this module interacts with the core SnRK2 signaling hub will be vital for a holistic understanding of the drought response network of pepper. Moreover, although MG132 treatment induced the accumulation of polyubiquitinated CaMAPK1 ladders *in vivo* (Figure S3A), whether these ubiquitinated species are generated predominantly by CaANKR1 or by additional E3 ligases remains unclear. Therefore, identifying downstream CaMAPK1 targets and additional regulators of CaMAPK1 stability will be important for understanding how this module is dynamically controlled during ABA‐ and drought‐responsive signaling.

In conclusion, our study demonstrates that CaMAPK1 positively regulates ABA‐mediated drought responses, whereas CaANKR1 negatively modulates this process by promoting CaMAPK1 ubiquitination and degradation. These findings reveal a previously uncharacterized mechanism by which a RING‐type E3 ligase modulates MAPK stability to fine‐tune ABA‐mediated drought signaling. This CaANKR1–CaMAPK1 module provides a molecular framework for engineering drought resilience through modulation of ubiquitin‐mediated MAPK stability.

## MATERIAL AND METHODS

### Plant materials

Pepper (*C*. *annuum* cv. Nock‐wang), *A. thaliana* (ecotype Col‐0), and tobacco (*Nicotiana benthamiana*) were used in this study. Seeds of pepper and tobacco were incubated in the dark at 28°C for 4 days. *Arabidopsis* seeds were sown on 1/2 Murashige and Skoog (MS) medium containing 0.8% agar and 1% sucrose (pH 5.7) and vernalized at 4°C for 2 days. All plants were grown under controlled environmental conditions at 24 ± 1°C with a 16 h light/8 h dark photoperiod, 60% relative humidity, and white fluorescent light (130 μmol m^−2^ sec^−1^). Soil composition was as described in previous studies (Cho et al., 2025). Briefly, a steam‐sterilized compost mixture containing peat moss, perlite, and vermiculite at a ratio of 5:3:2 was combined with sand and loam at a ratio of 1:1:1.

### Generation of *CaMAPK1* ‐OX transgenic *Arabidopsis* plants

The full‐length coding sequences (CDSs) of *CaMAPK1* and *CaANKR1* were cloned into the pCR8/GW/TOPO entry vector (Invitrogen, Waltham, MA, USA) and recombined into pGWB15 (Tsuyoshi et al., 2007) and p326GFP, respectively, using an LR recombination reaction. Thereafter, the constructs were introduced into *Agrobacterium tumefaciens* strain GV3101 and used to transform *A. thaliana* (Col‐0) via the floral dip method (Clough & Bent, 1998). Transgenic T1 plants were selected on 1/2 MS agar medium containing kanamycin (50 μg mL^−1^) or phosphinothricin (25 μg mL^−1^), depending on the construct.

### Virus‐induced gene silencing in pepper

Virus‐induced gene silencing (VIGS) was used to knock down the expression of *CaMAPK1* and *CaANKR1* in pepper, based on the procedure described in previous studies (Kim et al., 2025; Lim et al., 2026), with some modifications. The target sequences of CaMAPK1 (300 bp) were designed using the SGN VIGS tool (https://vigs.solgenomics.net/) and cloned into the TRV2 vector. *A. tumefaciens* harboring pTRV1 and the indicated pTRV2 constructs (pTRV2:00, pTRV2:*CaMAPK1*, and pTRV2:*CaANKR1*) were co‐infiltrated into the cotyledons of 2‐week‐old pepper. For double VIGS, four combinations were used: pTRV2:00 (empty vector control), pTRV2:*CaMAPK1*, pTRV2:*CaANKR1*, and pTRV2:*CaMAPK1*/*CaANKR1*. Two weeks after infiltration, the expression levels of *CaMAPK1* and *CaANKR1* were measured using RT‐PCR, and phenotype assays were also performed.

### Reverse transcription‐quantitative polymerase chain reaction analysis (RT‐qPCR)

Total RNA was isolated from plant tissues using TRI‐Reagent® (Molecular Research Center, Cincinnati, OH, USA) according to the manufacturer's instructions. RNA samples were treated with RNase‐free DNase I (New England Biolabs, Ipswich, MA, USA) to eliminate genomic DNA contamination. The concentration and quality of RNA were determined using a Synergy HTX Multi‐Mode Microplate Reader (BioTek Instruments, Winooski, VT, USA). cDNA was synthesized from 1 μg of total RNA using an iScript™ cDNA Synthesis Kit (Bio‐Rad Laboratories, Hercules, CA, USA). Quantitative real‐time PCR (RT‐qPCR) was performed using a CFX96 Touch Real‐Time PCR Detection System (Bio‐Rad Laboratories) with iQ™ SYBR Green Supermix (Bio‐Rad Laboratories). The relative expression levels of target genes were normalized using pepper Actin1 (*CaACT1*) or *Arabidopsis* Actin8 (*ACT8*) as internal controls. In RT‐qPCR analysis, the relative expression level of each gene was calculated using the ΔΔCt method (Livak & Schmittgen, 2001).

### Yeast two‐hybrid assay

The yeast two‐hybrid (Y2H) assay was performed as described previously (Lim et al., 2017). Briefly, the full‐length CDSs of *CaANKR1* and *CaMAPK1* were cloned into pGBKT7 [BD (DNA‐binding domain)] and pGADT7 [AD (activation domain)] vectors, respectively. The constructs were co‐transformed into yeast cells (*Saccharomyces cerevisiae* strain Y2HGold) using the lithium acetate method. Co‐transformants were initially selected on synthetic defined medium lacking leucine and tryptophan (SD/−Leu/−Trp). Individual colonies were subsequently cultured, and the cell density was adjusted to an OD_600_ of 0.5. Ten‐fold serial dilutions of each yeast culture were prepared, and 4 μl of each dilution was spotted onto SD/−Leu/−Trp medium and selective medium lacking adenine, histidine, leucine, and tryptophan (SD/−Ade/−His/−Leu/−Trp). The plates were incubated at 28°C, and yeast growth on the selective medium was assessed to determine the interaction between CaANKR1 and CaMAPK1.

### Expression of recombinant proteins and *in vitro* pull‐down assay

The recombinant proteins glutathione S‐transferase (GST)–tagged CaMAPK1 and maltose binding protein (MBP)–tagged CaANKR1 were expressed in bacterial cells and purified as previously described (Baek, Oh, et al., 2025; Park et al., 2015), with some modifications. For the *in vitro* pull‐down assay, GST–CaMAPK1 pre‐bound to glutathione Sepharose 4 Fast Flow resin (Cytiva, Marlborough, MA, USA) was incubated with MBP–CaANKR1 or MBP proteins expressed in *Escherichia coli* (BL21) under gentle rotation for 2 h at 4°C. After incubation, the resin was washed five times with STE buffer (20 mM Tris–HCl, pH 7.5, 10 mM NaCl, 5 mM EDTA) supplemented with 0.05% Triton X‐100. Each wash was performed for 10 min under gentle rotation. Bound proteins were detected using immunoblot analysis with anti‐GST (Santa Cruz Biotechnology, Dallas, TX, USA) and anti‐MBP (New England Biolabs) antibodies.

### Luciferase complementary interaction assay

The luciferase complementation imaging (LCI) assay was conducted as previously described (Baek, Oh, et al., 2025). Full‐length cDNAs of *CaMAPK1* and *CaANKR1* were cloned into pDEST14‐N‐LUC and pDEST14‐C‐LUC vectors as NLUC–CaMAPK1 and CLUC–CaANKR1, respectively. *A. tumefaciens* strain GV3101 carrying the indicated construct combinations, together with the p19 strain, was infiltrated into the epidermal cells of *N. benthamiana* leaves. After incubation for 3 days, the leaves were infiltrated with 0.1 mM d‐luciferin (BioVision, Milpitas, CA, USA) and incubated in the dark for 30 min. Subsequently, luciferase luminescence was detected using a Night‐Shade LB 985 imaging system (Berthold Technologies, Bad Wildbad, Germany), and images were processed and quantified using IndiGO software (version 3.0).

### Co‐immunoprecipitation assay

To analyze the interaction between CaMAPK1 and CaANKR1, 35S:3xFLAG–CaMAPK1 was co‐expressed with 35S:CaANKR1^CH^–GFP, an E3 ligase–deficient mutant (H338Y/C341S), or 35S:GFP in *N. benthamiana* leaves via *A. tumefaciens*–mediated infiltration using p19 strain cells. Three days after infiltration, the leaves were harvested, and protein extraction and immunoprecipitation (IP) assays were performed as previously described (Joo et al., 2024) with some modifications. Briefly, the harvested leaves were homogenized in protein extraction buffer containing 150 mM Tris–HCl (pH 7.5), 150 mM NaCl, 10 mM DTT, 1 mM EDTA, 10% glycerol, 2% PVPP, 0.2% Triton X‐100, and a 1× complete protease inhibitor cocktail. After insoluble debris was removed by centrifugation, the clarified protein extracts were incubated with anti‐GFP magnetic beads (ChromoTek GmbH, Planegg‐Martinsried, Germany) for 3 h at 4°C with gentle rotation. The beads were subsequently washed three times with washing buffer containing the same components as the protein extraction buffer but without PVPP. After each wash, the beads were collected using a magnetic separation rack. The input and immunoprecipitated protein fractions were subjected to immunoblot analysis using anti‐FLAG (Abcam, Cambridge, UK) and anti‐GFP (Santa Cruz Biotechnology, Dallas, TX, USA) antibodies.

### *In vitro* kinase assay

*In vitro* kinase activity assay was performed as described previously (Jeong et al., 2021), with some modifications. GFP‐tagged CaMAPK1 and its kinase‐dead mutant (K72N) were transiently expressed in *N. benthamiana* leaves, followed by IP using anti‐GFP magnetic beads. After washing, the bead‐bound proteins were subjected to an immune‐complex kinase assay in reaction buffer containing 1 mM CaCl_2_, 2.5 mM MgCl_2_, 2.5 mM MnCl_2_, 20 mM Tris–HCl (pH 7.5), 1 mM DTT, and 7.5 μCi [γ‐^32^P] ATP. The kinase reactions were incubated at 30°C for 3 h and terminated by adding 5× SDS‐PAGE sample buffer, followed by heating at 97°C. The reaction products were separated on a 7.5% SDS‐PAGE gel, and CaMAPK1 autophosphorylation was detected by ^32^P autoradiography using a Personal Molecular Imager (Bio‐Rad, Hercules, CA, USA).

### *In vitro* ubiquitination assay

For the *in vitro* ubiquitination assay, GST‐CaMAPK1 and MBP‐CaANKR1 or its CH mutant (MBP‐CaANKR1^CH^, H338Y/C341S) recombinant proteins were used. The assay procedure was performed as previously described with some modifications (Bae et al., 2024; Kim et al., 2025). Recombinant proteins (50 ng) were combined in various combinations with reaction buffer (50 mM Tris–HCl [pH 7.5], 5 mM MgCl_2_, 40 μM ZnCl_2_, 2 mM ATP, and 2 mM dithiothreitol [DTT]) supplemented with 100 ng recombinant human E1 (UBE1; Boston Biochemicals, Cambridge, MA, USA), 300 ng human E2 (UbcH5b; Enzo Life Sciences, Farmingdale, NY, USA), and 10 μg bovine ubiquitin (Sigma‐Aldrich, St. Louis, MO, USA) in a total volume of 40 μl. Afterward, the reaction mixtures were incubated at 28°C for 16 h, terminated by the addition of 5× SDS loading buffer, and analyzed by immunoblotting using anti‐GST, anti‐MBP, and K48‐linked polyubiquitin (Cell Signaling Technology, Danvers, MA, USA) antibodies.

### Cell‐free degradation assay

Crude extracts were prepared from 7‐day‐old *Arabidopsis* seedlings and leaves of pepper 2 weeks after agroinfiltration. Proteins were extracted using a buffer containing 25 mM Tris–HCl (pH 7.5), 10 mM MgCl_2_, 5 mM DTT, 0.1% Triton X‐100, 10 mM ATP, and 10 mM NaCl. GST–CaMAPK1 was incubated with crude extracts (50 μg total protein) for the indicated times, with or without MG132. Protein levels were analyzed by immunoblotting using an anti‐GST antibody.

### Phenotypic assay

The phenotypic assay of dehydration stress was conducted based on previously described methods with some modifications (Baek, Bae, et al., 2025; Lim et al., 2025). All assays were performed using pepper at the five‐leaf stage (2 weeks after *Agrobacterium* infiltration) and 3‐week‐old *Arabidopsis* plants. Dehydration tolerance was evaluated by subjecting plants to drought stress by withholding water for 14 days, followed by rewatering for 3 days. Survival rates were determined by counting plants that resumed growth after rewatering.

In transpiration water loss assays, the first and second leaves of pepper and the rosette leaves of 3‐week‐old *Arabidopsis* plants were detached and placed in Petri dishes under controlled conditions (40% relative humidity). Leaf fresh weights were measured at hourly intervals from 0 to 8 h, and water loss was calculated as a percentage of the initial fresh weight.

Leaf temperature was measured after spraying plants with 100 μM ABA, and thermal images were obtained 3 h post‐treatment using a T420 infrared camera (FLIR, Wilsonville, OR, USA). Temperatures were determined from the first and second fully expanded leaves of pepper and the rosette leaves of 3‐week‐old *Arabidopsis* plants, and average values were calculated using FLIR Tools+ software (version 5.2; FLIR).

Stomatal aperture assays were performed using abaxial epidermal peels prepared from pepper and *Arabidopsis* leaves. Samples were incubated in stomatal opening buffer (10 mM MES–KOH [pH 6.15], 50 mM KCl, and 10 μM CaCl_2_) under light conditions for 3 h, then transferred to fresh buffer containing 0, 10, or 20 μM ABA and incubated for an additional 2–3 h at 24°C. Stomata (>150 per sample) were randomly selected and observed using a Nikon Eclipse 80i microscope (Nikon, Tokyo, Japan), and stomatal apertures (width‐to‐length ratio) were measured using ImageJ software (National Institutes of Health, Bethesda, MD, USA).

For germination assays, 36 seeds were sown on 1/2 MS agar medium supplemented with either ABA (0 or 0.3 μM) or mannitol (0 or 400 mM). Germination rates were monitored daily for 4 days based on radicle emergence, and the percentage of seedlings with green cotyledons was determined 7 days after plating. Seedlings were grown vertically on 1/2 MS agar medium supplemented with 0 or 0.3 μM ABA for root elongation analysis. Also, to evaluate root growth during the post‐germinative stage, 3‐day‐old seedlings grown on 1/2 MS medium were transferred to 1/2 MS agar medium containing 0, 5, or 10 μM ABA. The plates were positioned vertically, and primary root lengths were measured 5 days after transfer.

## AUTHOR CONTRIBUTIONS

JC, CWL, BY, and SCL: conceptualization; JC, CWL, and DSK: methodology; JC and BY: data analysis; JC, CWL, and SCL: writing.

## CONFLICT OF INTEREST

The authors have no conflicts of interest to declare.

## Supporting information


**Figure S1.** Yeast two‐hybrid assay of CaANKR1 and CaMAPK family proteins. (A) The Y2HGold strain was co‐transformed with BD–CaANKR1 or the BD empty vector (negative control) and AD‐fused CaMAPK family proteins, then grown on SC–Leu–Trp (LW) medium as a control or SC–Ade–His–Leu–Trp (AHLW) medium for selection. Representative images were obtained after incubation at 28°C for 4 days.
**Figure S2.** Responses of *CaMAPK1*‐OX *Arabidopsis* plants to ABA during post‐germinative root growth and to mannitol during germination. (A, B) Post‐germinative root growth of wild‐type and *CaMAPK1*‐OX seedlings in response to ABA. Three‐day‐old seedlings grown on 1/2 MS medium were transferred to 1/2 MS agar medium supplemented with 0, 5, or 10 μM ABA. Representative images were obtained (A), and primary root length was measured 5 days after transfer (B) (*n* = 18 seedlings per genotype). (C) Germination rates of WT and transgenic lines (#6 and #9) in response to mannitol. Seeds of each line were sown on 1/2‐strength Murashige and Skoog (1/2 MS) agar plates containing either 0 or 400 mM mannitol. Germination was scored by counting seeds showing radicle emergence at the indicated time points after plating. (D) Cotyledon greening of WT and transgenic lines in response to mannitol. Seeds were germinated on 1/2 MS agar plates supplemented with 0 or 400 mM mannitol. Seven days after sowing, the percentage of seedlings with green cotyledons was determined (*n* = 72 seeds per genotype). All data represent the mean ± SE of three independent biological replicates. Statistical analyses were performed using two‐way ANOVA followed by Tukey's post hoc test (*P* < 0.05). Different letters indicate significant differences among genotypes within the same treatment, whereas genotypes sharing the same letter are not significantly different.
**Figure S3.** Ubiquitin‐dependent degradation of CaMAPK1 via the 26S proteasome. (A) Degradation of GFP‐tagged CaMAPK1 proteins in the leaves of *Nicotiana benthamiana* plants. At 2 days after agroinfiltration, the leaves were infiltrated with 50 μM cycloheximide (CHX) and/or 50 μM MG132. Immunoblot analysis was performed using anti‐GFP (top) and anti‐UBQ1 (middle) antibodies, and Coomassie Brilliant Blue (CBB) staining was used as a loading control (bottom).
**Figure S4.** RT‐PCR analysis of *CaANKR1* expression in *Arabidopsis* and cell‐free degradation assay of GST‐CaMAPK1 in *CaANKR1*‐OX plants. (A) RT‐PCR analysis of *CaANKR1* expression in wild‐type (WT) and *CaANKR1*‐OX *Arabidopsis* lines expressing intact or CH mutant forms. *ACT8* was used as an internal control. (B) Cell‐free degradation assay for CaMAPK1 in *CaANKR1*‐OX plants expressing intact (I) or CH mutant (C) forms. GST–CaMAPK1 protein was incubated with crude extracts prepared from WT or *CaANKR1*‐OX *Arabidopsis* plants for 0, 1, or 2 h with MG132 treatment at 2 h. Immunoblot analysis was performed using anti‐GST antibody (top). Coomassie Brilliant Blue (CBB) staining indicates equal loading of crude extract (bottom). The relative intensities of GST–CaMAPK1 fusion proteins were quantified using ImageJ software (version 1.52a). Protein levels were normalized to the initial level (0 h), which was set to 1.0. Statistical significance was determined using Student's *t*‐test (*P* < 0.05).
**Figure S5.** Verification of gene silencing efficiency in double VIGS pepper. RT‐qPCR analysis of *CaMAPK1* and *CaANKR1* expression levels in the leaves of control (pTRV2:00), single‐silenced (pTRV2:*CaMAPK1* and pTRV2:*CaANKR1*), and double‐silenced (pTRV2:*CaMAPK1*/*CaANKR1*) pepper. *CaACT1* was used as an internal control. Data represent the mean ± SE of three independent biological replicates. Statistical analyses were performed using two‐way ANOVA followed by Tukey's post hoc test (*P* < 0.05). Different letters indicate significant differences among genotypes within the same treatment, whereas genotypes sharing the same letter are not significantly different.
**Table S1.** Primers used in this study.

## Data Availability

All data are available in the manuscript or its Supplementary Files. The raw data supporting the findings of this study are available from the corresponding author upon reasonable request.
