# Supplementary material for: Pepper RING‐type E3 ligase, CaANKR1, negatively regulates drought tolerance by modulating CaMAPK1 stability
Source: Plant J. 2026 Oct 14;128(1):e71181. doi: 10.1111/tpj.71181 (PMC13669525; doi:10.1111/tpj.71181)
Supplement: Supplementary file 1 [file TPJ-128-0-s001.pdf]

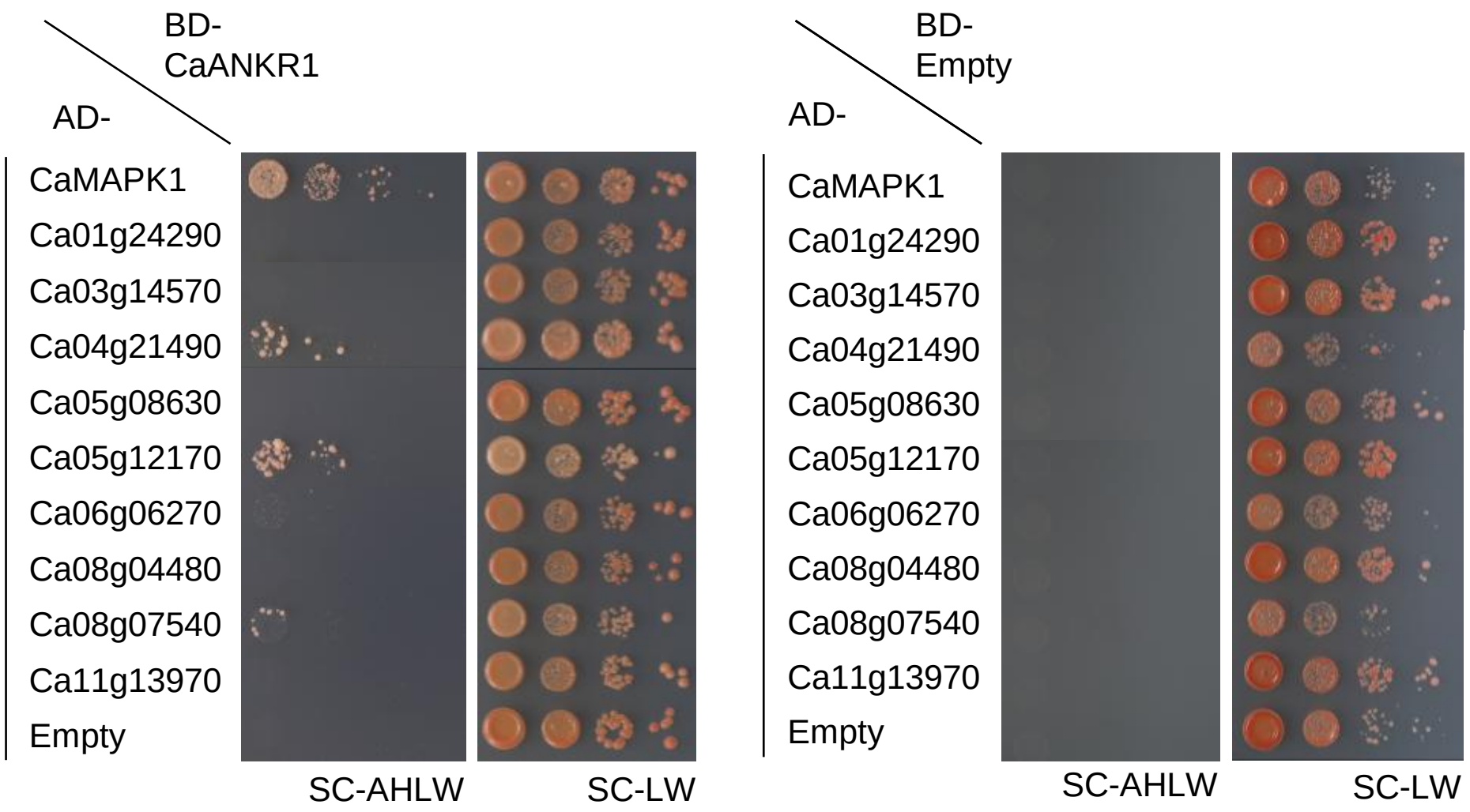

Figure S1. Yeast two-hybrid assay of CaANKR1 and CaMAPK family proteins. (A) The Y2HGold strain was co-transformed with BD–CaANKR1 or the BD empty vector (negative control) and AD-fused CaMAPK family proteins, then grown on SC–Leu–Trp (LW) medium as a control or SC–Ade–His–Leu–Trp (AHLW) medium for selection. Representative images were obtained after incubation at 28°C for 4 days.

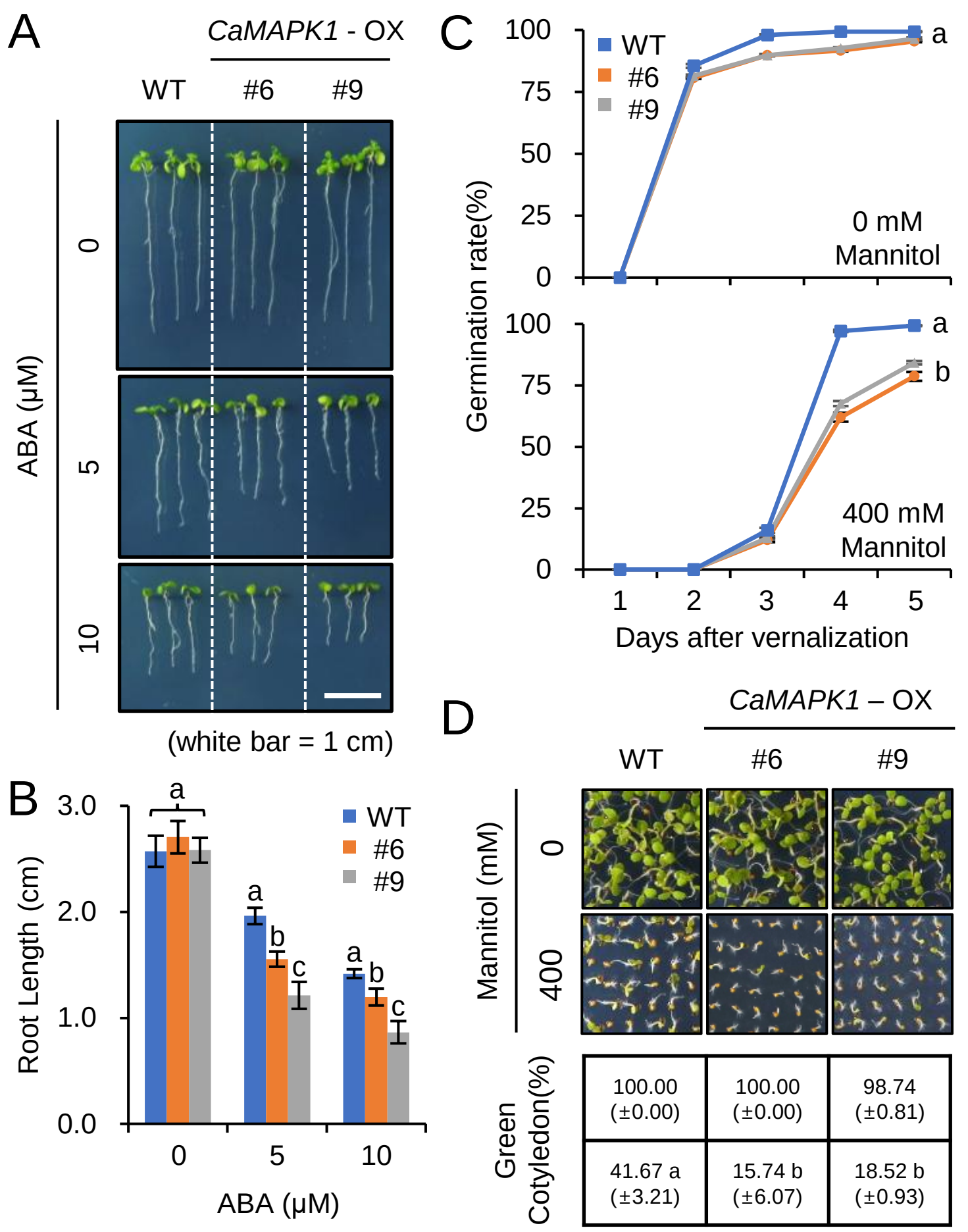

Figure S2. Responses of *CaMAPK1*-OX *Arabidopsis* plants to ABA during post-germinative root growth and to mannitol during germination. (A, B) Post-germinative root growth of wild-type and *CaMAPK1*-OX seedlings in response to ABA. Three-day-old seedlings grown on 1/2 MS medium were transferred to 1/2 MS agar medium supplemented with 0, 5, or 10 μM ABA. Representative images were obtained (A), and primary root length was measured 5 days after transfer (B) (*n* = 18 seedlings per genotype). (C) Germination rates of WT and transgenic lines (#6 and #9) in response to mannitol. Seeds of each line were sown on 1/2-strength Murashige and Skoog (1/2 MS) agar plates containing either 0 or 400 mM mannitol. Germination was scored by counting seeds showing radicle emergence at the indicated time points after plating. (D) Cotyledon greening of WT and transgenic lines in response to mannitol. Seeds were germinated on 1/2 MS agar plates supplemented with 0 or 400mM mannitol. Seven days after sowing, the percentage of seedlings with green cotyledons was determined (*n* = 72 seeds per genotype). All data represent the mean ± SE of three independent biological replicates. Statistical analyses were performed using two-way ANOVA followed by Tukey's post hoc test ($P < 0.05$). Different letters indicate significant differences among genotypes within the same treatment, whereas genotypes sharing the same letter are not significantly different.

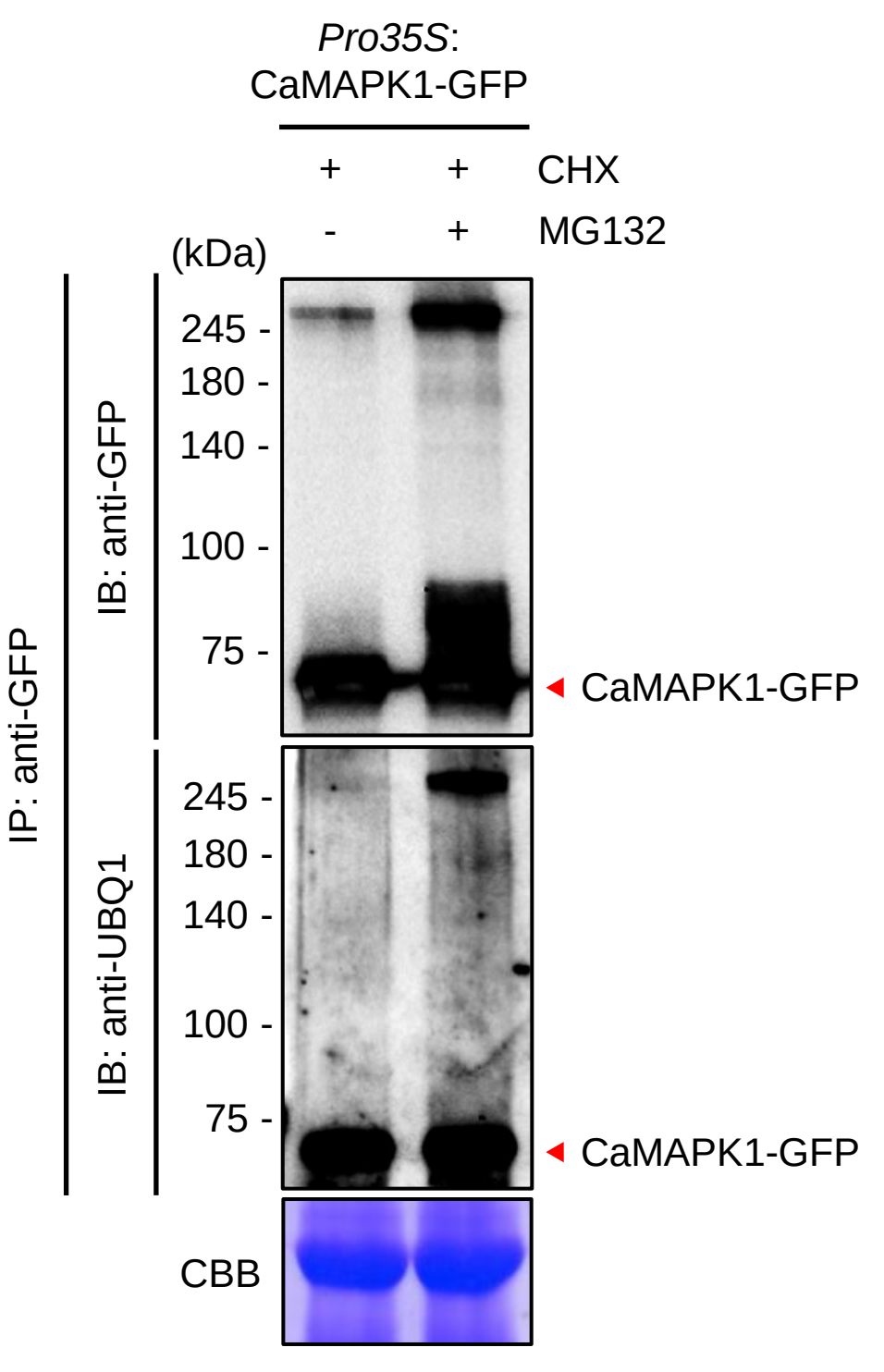

Figure S3. Ubiquitin-dependent degradation of CaMAPK1 via the 26S proteasome. (A) Degradation of GFP-tagged CaMAPK1 proteins in the leaves of *N. benthamiana* plants. At 2 days after agroinfiltration, the leaves were infiltrated with 50 μM cycloheximide (CHX) and/or 50 μM MG132. Immunoblot analysis was performed using anti-GFP (top) and anti-UBQ1 (middle) antibodies, and Coomassie Brilliant Blue (CBB) staining was used as a loading control (bottom).

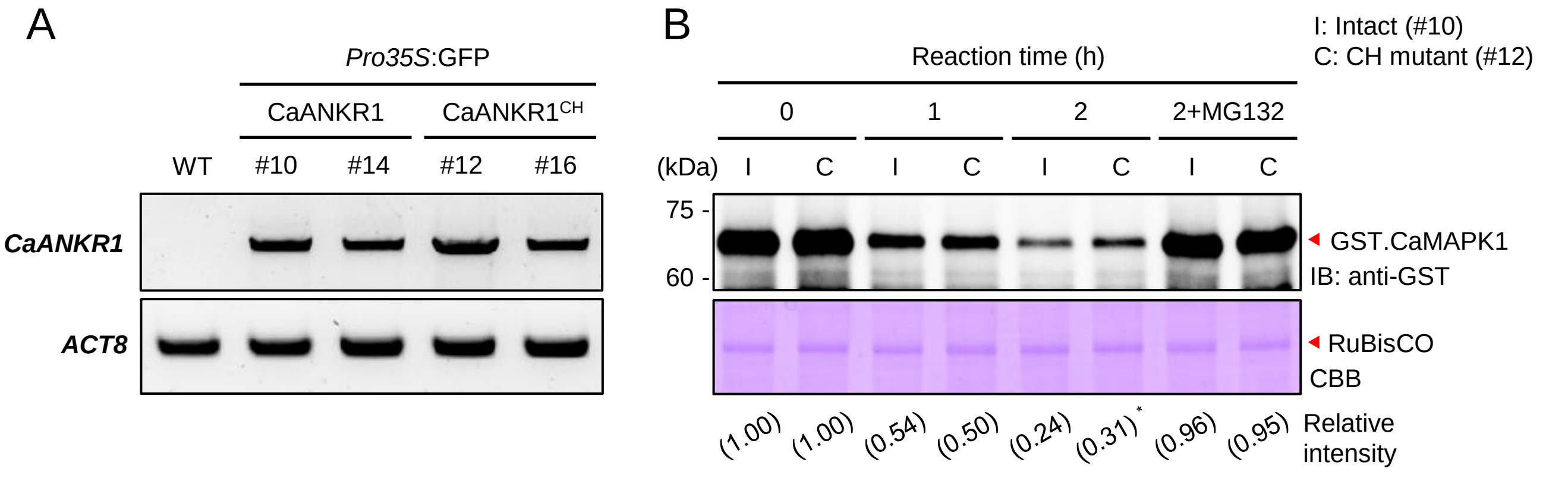

Figure S4. RT-PCR analysis of *CaANKR1* expression in *Arabidopsis* and cell-free degradation assay of GST-CaMAPK1 in *CaANKR1*-OX plants. (A) RT-PCR analysis of *CaANKR1* expression in wild-type (WT) and *CaANKR1*-OX *Arabidopsis* lines expressing intact or CH mutant forms. *ACT8* was used as an internal control. (B) Cell-free degradation assay for CaMAPK1 in *CaANKR1*-OX plants expressing intact (I) or CH mutant (C) forms. GST–CaMAPK1 protein was incubated with crude extracts prepared from WT or *CaANKR1*-OX *Arabidopsis* plants for 0, 1, or 2 h with MG132 treatment at 2 h. Immunoblot analysis was performed using anti-GST antibody (top). Coomassie Brilliant Blue (CBB) staining indicates equal loading of crude extract (bottom). The relative intensities of GST–CaMAPK1 fusion proteins were quantified using ImageJ software (version 1.52a). Protein levels were normalized to the initial level (0 h), which was set to 1.0. Statistical significance was determined using Student's *t*-test ($P < 0.05$).

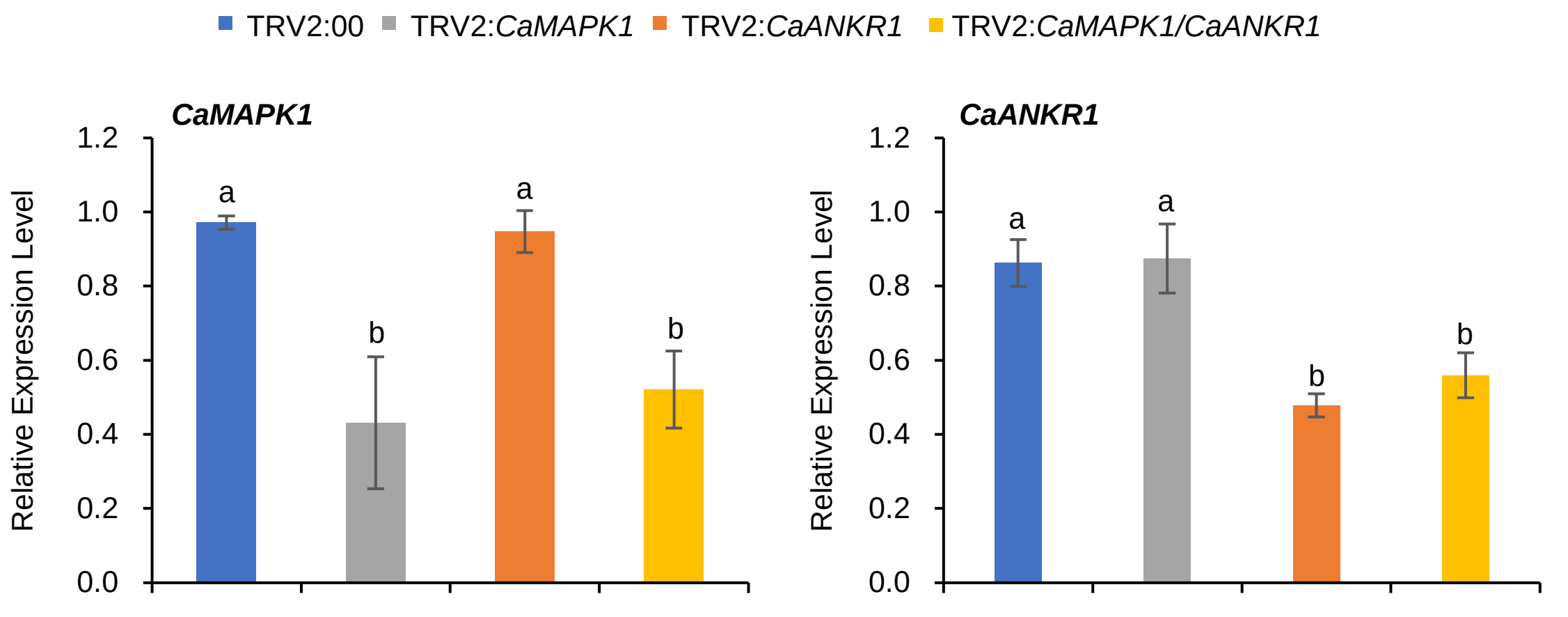

Figure S5. Verification of gene silencing efficiency in double VIGS pepper. RT-qPCR analysis of *CaMAPK1* and *CaANKR1* expression levels in the leaves of control (pTRV2:00), single-silenced (pTRV2:*CaMAPK1* and pTRV2:*CaANKR1*), and double-silenced (pTRV2:*CaMAPK1*/*CaANKR1*) pepper. *CaACT1* was used as an internal control. Data represent the mean ± SE of three independent biological replicates. Statistical analyses were performed using two-way ANOVA followed by Tukey's post hoc test ($P < 0.05$). Different letters indicate significant differences among genotypes within the same treatment, whereas genotypes sharing the same letter are not significantly different.

Supplementary Table 1. List of primers used in this study.

Primer name	Primer sequence (5'-3')
**For cloning**	
CaMAPK1 CDS	Forward: ATGGCCTTAGTTCGAGACCGC
	Reverse: TCAGTTTTGCCCGTTACTCCGT
CaMAPK1 noST	Forward: CGGAGTAACGGGCAAAACAAGGGCGAATTCG
	Reverse: CGAATTCGCCCTTGTTTTGCCCGTTACTCCG
CaMAPK1 VIGS	Forward: TCTAGAATGGCTAATCAAGCCGGCG (XbaI)
	Reverse: CTCGAGTTCGTGATCCATATGCCGAAGTA (XhoI)
CaANKR1 CDS	Forward: ATGGGTCAGAAACTCAGTTGTGGAA
	Reverse: TCAGTCAAGAATTATTGGCTTATCA
**For qRT-PCR**	
CaMAPK1	Forward: AGAACATTGAGAGAAATTAAGCTACTTC
(CA01g02140)	Reverse: AATCTTGAGGTCGCAATTTGCAT
CaANKR1	Forward: ATCATCCTCAGAGCTGCTGGAGC
(CA06g24570)	Reverse: TTAAGGATCTTAAGCGTGGTTATGT
CaACT1	Forward: AGATTAGTTCAAGAACGTGAATTGG
(CA12g08730)	Reverse: GACGTGACCTAACTGATAACCTGAT
